# Familial forms of nephrotic syndrome

**DOI:** 10.1007/s00467-008-1051-3

**Published:** 2010-02-01

**Authors:** Gianluca Caridi, Antonella Trivelli, Simone Sanna-Cherchi, Francesco Perfumo, Gian Marco Ghiggeri

**Affiliations:** 1 Laboratory on Physiopathology of Uremia, G. Gaslini Children Hospital, Genoa, Italy; 2 Department of Medicine, Division of Nephrology, Columbia University College of Physicians and Surgeons, New York, NY USA; 3 Division of Nephrology, Dialysis and Transplantation, G. Gaslini Children Hospital, Largo G. Gaslini, 5, 16148 Genova, Italy

**Keywords:** Molecular genetics, Nephrin, Nephrotic syndrome, Podocin, Podocytes

## Abstract

The recent discovery of genes involved in familial forms of nephrotic syndrome represents a break-through in nephrology. To date, 15 genes have been characterized and several new loci have been identified, with a potential for discovery of new genes. Overall, these genes account for a large fraction of familial forms of nephrotic syndrome, but they can also be recognized in 10–20% of sporadic cases. These advances increase diagnostic and therapeutic potentials, but also add higher complexity to the scenario, requiring clear definitions of clinical, histopathological and molecular signatures. In general, genetic forms of nephrotic syndrome are resistant to common therapeutic approaches (that include steroids and calcineurin inhibitors) but, in a few cases, drug response or spontaneous remission suggest a complex pathogenesis. Finally, syndromic variants can be recognized on the basis of the associated extra-renal manifestations. In this educational review, clinical, histological and molecular aspects of various forms of familial nephrotic syndrome have been reviewed in an attempt to define a rational diagnostic approach. The proposed model focuses on practical and economic issues, taking into consideration the impossibility of using genetic testing as starting diagnostic tool. The final objective of this review is to outline a diagnostic flow-chart for clinicians and geneticists and to generate a rational scheme for molecular testing.

## Introduction

With an estimated [1] annual incidence of between 2.0 and 2.7 cases per 100,000 children in the USA and a cumulative prevalence of 16 per 100,000 [2, 3], idiopathic nephrotic syndrome (NS) is the prevailing glomerular disease in children. It is defined as the association of gross proteinuria with hypoalbuminemia, edema and hyperlipidemia, a condition that usually requires prolonged and combined treatments, and one that may recur over years. Nephrotic syndrom appears to be a clinically heterogeneous disease characterized by different histological variants [4–6] and genetic determinants [7–9]. The recent discovery of genes involved in idiopathic NS represents a milestone in nephrology. Most of these genes code for structural elements of the slit diaphragm and of podocyte cytoskeleton (*NPHS1*, *NPHS2*, *CD2AP*, *TRCP6*, *ACTN4*); others are expressed in the glomerular basement membrane (*LAMB2*) and in mitochondria (*COQ2*) (see Table 1). Yet another group includes transcription factors necessary for normal podocyte function and development (*WT1*, *LMX1B)*. A thorough definition of histopathological features is crucial for a correct genetic approach to idiopathic NS since, in many cases, a genotype–phenotype correlation can help to address the underlying molecular defect. Nevertheless, there is wide overlap between different forms and cases of uncommon associations, such as the presence of diffuse mesangial sclerosis in patients with *NPHS2* mutations or focal glomerulosclerosis in patients with variants in the *PLCE1* gene.

**Table 1:** Principle genes involved in familial nephrotic syndrome and in associated syndromes

Syndromes	Gene	Locus	Protein	Inheritance	Prevalent histology	OMIM number
Familial nephrotic syndrome						
Nephrotic syndrome, Finnish type	*NPHS1*	19q13.1	Nephrin	AR	DMS, microcysts	602716
Nephrotic syndrome, steroid-resistant type 2	*NPHS2*	1q25–31	Podocin	AR	FSGS	604766
Nephrotic syndrome, steroid-resistant type 3	*PLCE1*	10q23	Phospholipase C epsilon 1	AR	DMS	610725
Denys–Drash syndrome	*WT1*	11p13	Wilms tumor 1 gene	AD	DMS	194080
Frasier syndrome	*WT1*	11p13	Wilms tumor 1 gene	AD	FSGS	136680
Focal segmental glomerulosclerosis type 1	*ACTN4*	19q13	Alpha-Actinin 4	AD	FSGS	603278
Focal segmental glomerulosclerosis type 2	*TRPC6*	11q21–22	Transient receptor potential cation channel, homolog of 6	AD	FSGS	603965
Focal segmental glomerulosclerosis type 3	*CD2AP*	6p12	CD2-associated protein	AR/AD	FSGS	607832
Associated syndromes						
Schimke immuno-osseous dysplasia	*SMARCAL1*	2q34–q36	SWI/SNF-related, matrix-associated, actin-dependent regulator of chromatin, subfamily a-like protein 1	AR	FSGS	242900
Pierson syndrome	*LAMB2*	3p21	Laminin beta 2	AR	FSGS	609049
COQ2 deficiency	*COQ2*	4q21–q22	Parahydroxybenzoate-polyprenyltransferase	AR	FSGS, Collapsing	607426
Leigh syndrome	*PDSS2*	6q21	Decaprenyl diphosphate synthase, subunit 2	AR	FSGS, Collapsing	607426
AMRF syndrome (Action myoclonus-renal failure syndrome)	*SCARB2*/*LIMP2*	4q13–q21	Scavenger receptor class B, member 2	AR	FSGS	254900

*AR* Autosomal recessive; *AD* autosomal dominant; *DMS* diffuse mesangial sclerosis; *FSGS* focal segmental glomerulosclerosis; *OMIM* Online Mendelian Inheritance in Man database; *SWI/SNF* swItch/sucrose nonfermentable nucleosome remodeling complex

**Fig. 1:**
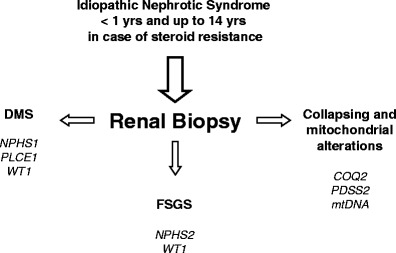
Diagnostic flow-chart in children with early onset nephrotic syndrome (<1 year) or in patients <14 years demonstrating steroid resistance. Steroid resistance was defined by lack of anti-proteinuric effect after 45 days with prednisone 2 mg/kg and three pulses with methyl-prednisolone 10 mg/kg. *DMS* Diffuse mesangial sclerosis, *FSGS* focal segmental glomerulosclerosis, *NPHS1* nephrin gene, *NPHS2* podocin gene, *PLCE1* phospholipase C epsilon 1 gene, *WT1* Wilm’s tumor 1 gene, *COQ2* para-hydroxybenzoate-polyprenyl-transferase gene, *PDSS2* decaprenyl diphosphate syntase gene, *mtDNA* mitochondrial DNA

Even though the review of the histopathological aspects of idiopathic NS is not within the scope of our educational review and readers should refer to specialized papers [10–12], a few remarks could facilitate comprehension. A distinction is generally accepted between a first group that includes minimal change disease (MCD), mesangial proliferation with deposition of IgM (MesIgM) and focal segmental glomerulosclerosis (FSGS) as different stages of the same entity and a second one only represented by diffuse mesangial sclerosis (DMS) [13]. It is important to stress that FSGS is not a single condition but includes five histological variants, i.e. collapsing FSGS, tip lesion, cellular variant, perihilar lesions and a final one without specified alterations. Recognizing these sub-categories has diagnostic, therapeutic and prognostic implications. Collapsing FSGS is, for example, usually associated with human immunodeficiency virus (HIV) infection and with mitochondrial defects, while the unspecified variant is often associated with *NPHS2* mutations. In the diagnostic flow-chart used at Istituto Giannina Gaslini (Fig. 1), the definition of renal histopathology plays a central role for addressing the molecular approach. This scheme is applied to all children presenting with NS in the first year of life and in children between two and 14 years of age with steroid resistance.

## Genetic variant associated with FSGS/MesIgM/MCD

There are several genes causing isolated FSGS/MesIgM/MCD with both autosomal recessive (AR) (*NPHS2)* and dominant (AR) (*ACTN4*, *TRPC6*) inheritance and other modes (such as in the case of *CD2AP*), which are currently under investigation. Overall, recessive forms are more frequent than dominant traits, and *NPHS2* is by far the gene most frequently implicated in FSGS.

### *NPHS2/*podocin (AR) (OMIM#604766)

The *NPHS2* gene codes for podocin, which is a raft-associated component of the glomerular foot-process membrane where the protein is localized at the insertion of the slit-diaphragm [8, 14, 15] (GenBank NM_014625; NP_055440). Podocin belongs to the stomatin domain family proteins, which are uniquely expressed in the kidney glomeruli where they interact with nephrin and CD2AP. Mutations in the *NPHS2* gene have been initially identified in families with AR-FSGS, where they account for most familial nephrotic syndromes with recessive inheritance [8]. Extensive data have also been published on *NPHS2* mutational screenings in patients with sporadic NS with recessive inheritance [16–21], most of whom are children with steroid resistance. Overall, *NPHS2* mutations have been reported to account for a significant proportion of all nephrotic patients, corresponding roughly to 45–55% of familial forms and 8–20% of sporadic disease, with variations according to the different patient cohorts and the different sub-phenotypes studied. However, the actual incidence is probably much higher if only patients with a poor response to steroids and/or showing a pathological picture of FSGS are considered. More than 40 *NPHS2* mutations have been reported to date that involve the whole length of the gene; these determine every kind of alteration, including deletions, splice site, missense and nonsense variants. The onset of proteinuria has been reported to occur at different ages, but generally before the 14^th^ year of life. A few cases of congenital NS have been described, including two families with double homozygous p.R168H and p.P20L mutations [16, 19]. With respect to a possible genotype–phenotype correlation, some inferences can be drawn: (1) p.R138Q appears to be associated with early onset NS (at 12 ± 3 months of age in 15 patients) whereas p.V180M and p.R238S are associated with a late onset form (at 129 ± 12 months in seven patients); (2) carriers of the association of p.R229Q variant with other *NPHS2* mutations present late onset proteinuria, generally between the first and second decades of life; (3) carriers of *NPHS2* mutations generally do not respond to drugs commonly used in NS, with the exception of four partial responders to cyclosporin described by Ruf and colleagues [18]; (4) progression to end stage renal failure is rapid in all cases and usually occurs within the first decade of life [21]. Data on histopathological phenotype are available for 48 patients with homozygous or compound heterozygous *NPHS2* mutations and for 15 with p.R229Q associated with another *NPHS2* mutation. Focal segmental glomerulosclerosis was the prevailing glomerular lesion in 39 patients of the former group and in 15 of the p.R229Q/heterozygous *NPHS2* cohort. Ten patients had milder renal changes epitomized by mesangial proliferation with IgM deposition in eight cases and minimal changes nephropathy in two. Finally, a few cases presented DMS and one biopsy showed a nonspecific C3 deposition [17, 18]. Therefore, the variability of renal histopathological phenotype associated with *NPHS2* mutations limits in some way the possibility of a simple diagnosis based on two diagnostic tests (histology and *NPHS2* sequence). A major confounder is represented by the presence, even if unusual, of DMS, which can complicate the molecular approach.

### *TRPC6/*transient receptor potential cation channel, homolog of 6 (AD) (OMIM#603965)

The *TRCP6* gene codes for a glomerular slit diaphragm-associated channel that is required for normal renal function. It is implicated in several biological functions, but mainly in intracellular ion homeostasis and calcium entry into cells. *TRCP6* was first localized to chromosome 11q21–22 by Winn and colleagues [22] and then identified as a cause of FSGS in a large pedigree study from New Zealand [23]. Subsequently, Reiser and colleagues [24] confirmed *TRPC6* mutations in another five unrelated families of different ancestry in which mutations were inherited following an AR mode with reduced penetrance. Five of the described mutations predicted an amino acid change—one in the ankyrin domain, two in the N-terminal intracellular domain and two in the C-terminal intracellular domain—and only one generated a stop codon at the C terminus. Functional studies of three families showed that *TRPC6* mutations increased the influx of calcium into cells. One likely explanation is that increased intracellular calcium modifies the contractile structure of podocytes or, alternatively, high calcium may cause podocytopenia through apoptosis, detachment or lack of proliferation. Clinical features related to *TRCP6* mutations are rather homogeneous since the first symptoms occur in the third–fourth decade of life and usually consist of severe proteinuria that is always associated with FSGS. Progression to end stage renal failure has been reported in 50% of the patients [25].

### *ACTN4/*Alpha-Actinin-4 (AD) (OMIM#603278)

Mutations of *ACTN4*, the gene coding for α-Actinin 4, cause a rare form of familial autosomal-dominant NS with reduced penetrance. The function of this protein is to crosslink actin filaments and connect them to the glomerular basement membrane. To date, six families and sporadic cases have been reported [9, 26, 27], all showing a mild increase in urine protein excretion during adolescence or early adulthood and slow progression to end stage renal failure in some affected individuals. In all cases described to date, the *ACTN4* mutations were in close proximity (exon 8) and predicted an amino acid change in the actin-binding domain. This finding suggests the existence of a mutational hot-spot that would facilitate, if confirmed in a larger population, the molecular work-up. Classical FSGS is the most frequent histopathologic lesion related to *ACTN4* mutations, but one child showing the collapsing variant associated with rapidly progressing NS in early childhood has also been reported [27].

### *CD2AP/*CD2-associated protein (AR/AD) (OMIM#607832)

CD2-associated protein (*CD2AP*) is an adapter molecule originally identified as a ligand for the T cell-adhesion protein CD2. It is an 80-kDa cytoplasmic protein expressed in all tissues except the brain. In the kidney, CD2AP is localized to podocytes. The results of experiments in mice with targeted disruption of CD2AP [28] and functional studies [29] strongly suggest that *CD2AP* is a putative gene in inherited NS. A unique patient with a homozygous truncating mutation of *CD2AP* (stop codon at amino acid position 612) has been recently described by Löwik and colleagues [30]. This patient had developed early onset NS with a histological pattern sharing some features of FSGS and diffuse mesangial sclerosis patterns. A few other patients with NS and heterozygous mutations of *CD2AP* have also been reported. In a first report based on the results of their study on 45 African-Americans, Kim et al. [31] described a heterozygous nucleotide variant resulting in aberrant *CD2AP* splicing in two patients with idiopathic FSGS and HIV infection. Three heterozygous mutations (c.944A>T; c.1161A>G; c.1573delAGA) producing amino acid changes (p.K301M, p.T374A) or a deletion in functional domains (p.delG525), respectively, were found in three unrelated patients in Italy [32], and the functional consequences of these were studied in two. One mutation (p.K301M) resulted in defective CD2–CD2AP interaction and clustering; the other (c.1573delAGA) was associated with the down-regulation of *CD2AP* and podocin glomerular expression. All three patients presented overt proteinuria with steroid NS at variable ages (one in adulthood; the other two in childhood), and FSGS was the corresponding histopathological finding. Haplo-insufficiency of CD2AP due to heterozygous mutations may confer susceptibility to glomerular disease and integrity, as suggested by experiments in *CD2AP* heterozygous knockout mice [31].

## Genetic variants associated with isolated DMS

Diffuse mesangial sclerosis is a common histopathological feature in renal biopsies of patients with congenital NS. It is characterized by mesangial expansion and sclerosis that evolve towards obliteration of the capillary lumen and contraction of the glomerular tuft. Typically, there is no cell proliferation in DMS; rather, there is an accumulation of collagen-like fibrils in the mesangium and glomerular basement membrane, causing thickening. The immunofluorescence is usually negative. Diffuse mesangial sclerosis does not represent a single entity, but is associated with several syndromic disorders, such as Denys–Drash, Galloway–Mowat, Pierson, carbohydrate-deficient glycoprotein syndrome type I (see below) and even with viral infections. In some cases, such as in NS of the Finnish type, DMS is associated with diffuse microcysts and has been described separately from the pure glomerular form.

### *PLCE1/*phospholipase C epsilon (AR) (OMIM #610725)

Phospholipase C epsilon is a cytoplasmatic enzyme that is required during podocyte maturation. *PLCE1* gene mutations have been recently identified in association with congenital NS in several families from Turkey and one from Israel, and *PCLE1* is currently considered to be a major gene responsible for NS during the first year of life [33]. Children lacking PCLE1 for truncating mutations present DMS that is due to a complete block of glomerular maturation at the 23rd gestational week. More recently, *PCLE1* truncating mutations were identified in ten of 35 children with isolated DMS (28.3%), suggesting that *PLCE1* mutations are a major cause of DMS [34]. A less severe course characterized by FSGS and slower progression to end stage renal failure has been reported in patients with *PLCE1* missense mutations, implying a genotype–phenotype correlation and suggesting the clinical relevance of genetic testing [35]. It has also been proposed that renal histopathological patterns reflect the different kidney developmental stages at which PCLE1 activity is required. According to this hypothesis, DMS results from a developmental arrest due to truncating *PLCE1* mutations, whereas FSGS is the phenotype arising from low-level or dysfunctional *PLCE1* in the case of less severe molecular defects [35]. A further unexpected finding was a complete response to steroid treatment in a girl with a homozygous truncating *PLCE1* mutation [35]. It seems reasonable to propose that, in this case, steroids could have activated some cellular response downstream of *PLCE1* that substitutes phospholipase in the immunosuppressive effect.

## Congenital nephrosis of the Finnish type

### *NPHS1*/nephrin (AR) (OMIM#256300)

Nephrin is probably the most structurally important protein in podocytes where it represents the major constituent of the slit-diaphragm at the regulation site for ultra-filtration of proteins. It is encoded by *NPHS1*, a 26-kb gene located on chromosome 19q13.1 [36]. Mutations in *NPHS1* cause an AR disorder characterized by a congenital NS that is common in Finland; for this reason it is called Finnish nephropathy (FN). Finnish nephropathy is also present outside Finland and, in a recent survey of 80 sporadic cases [37], *NPHS1* mutations accounted for 22.5% of nephrotic cases in the first year of life and was second to only *NPHS2* as a causal factor of NS. More than 90 *NPHS1* mutations have been identified so far [38, 39]. In Finland, most patients have mutations in exons 2 and 26, defined as Fin_major_ (80% of cases) and Fin_minor_, respectively. These mutations cause truncated proteins of 90 and 1109 amino acids, respectively, leading to the absence of nephrin in the slit-diaphragm. A variety of mutations (missense, nonsense, splice, deletions and insertions) have been reported outside Finland in association with variable phenotypes [7, 40]. Missense mutations, that are frequently observed outside Finland cause a milder phenotype (see below). Molecular studies demonstrated an altered subcellular distribution of these mutant nephrins, which accumulate in endoplasmic reticulum and do not reach the cell membrane [41, 42].

Renal histopathology in FN biopsies at 3 months of life showed mixed glomerular and tubulointerstitial lesions. Glomerular lesions vary from mesangial hypercellularity to glomerular sclerosis and/or diffuse mesangial sclerosis. Tubular aspects are more characteristic and include diffuse cystic dilatations of proximal and distal tubules (also known as microcysts); interstitial fibrosis and inflammation are usually present and increase with age. Immunofluorescence is always negative and electron microscopy shows foot process effacement and villous transformation [43, 44]. Nephrin staining provides a definitive diagnosis in those cases with truncating mutations since the slit diaphragm, that is mainly composed of nephrin, may be absent in non-sclerotic glomeruli. This is particularly important for a differential diagnosis between FN and other congenital nephrotic syndromes since nephrin staining can help to detect the underlying defect [45].

The disease is characterized by severe proteinuria often present ‘in utero’ and almost always appearing in the first year of life. Steroid and calcineurin inhibitor resistance and the requirement for albumin infusion and parenteral nutrition characterize this life-threatening condition. Disease course is rapidly progressive, and end stage renal failure generally occurs within the first decade of life. In rare cases, FN may have a mild phenotype. Patrakka and colleagues [46] described a patient with compound heterozygous Fin_minor_ and missense mutations who responded to indomethacin and angiotensin-converting enzyme (ACE) inhibitors, and another four cases with missense mutations associated with remittent proteinuria have been reported in Japan [47] and in Germany [39].

A recent report by Philippe and colleagues [42] emphasizes the occurrence of mild clinical phenotypes. They described a good clinical outcome in six patients who actually carried at least one severe mutation that in most cases determined a truncating protein. Histology showed MCD in five patients and FSGS in one patient; clinical follow-up was characterized by a good anti-proteinuric effect of corticosteroids alone or in association with cyclosporin. One child had normal renal function at 6 years of age despite the absence of therapy.

The co-existence of *NPHS1* and *NPHS2* mutations was observed in a few patients in association with congenital FSGS or FN [48]. This result underlines a functional link between nephrin and podocin that is also supported by cellular studies on their interaction [49]. The results of molecular studies suggested that, in order to produce congenital NS, a triple hit is required, with a homozygous event in one gene and the third mutation acting as modifier of the others. The renal phenotype in carriers of this defect ranges from FN to congenital FSGS, thereby supporting the theory that these disorders can be regarded as part of a spectrum, with the resulting effect being determined by the molecular background. These observations also highlight that, in the presence of FSGS and/or DMS, the molecular diagnosis should incorporate the analysis of both *NPHS2* and *NPHS1* according to a flow chart that starts with *NPHS1* in the case of FN, and *NPHS2* in the case of FSGS.

## Syndromic forms

### Denys–Drash (OMIM#194080) and Frasier syndromes (OMIM#136680)

Denys–Drash (DDS) and Frasier syndromes (FS) are caused by *WT1* gene mutations. This gene codes for a transcriptional factor (Wilms’ tumor suppressor protein) of the zinc-finger protein family that is involved in kidney and gonadal development. It consists of ten exons and is localized on chromosome 11q13. It leads to four different isoforms, depending on alternative splicing [50]. After birth, WT1 protein expression is restricted to renal podocytes where it probably contributes to maintaining cellular differentiation [51]. Germline heterozygous *WT1* mutations have been extensively reported in the literature as the cause of the two predominant sporadic phenotypes, i.e. DDS and FS, which are characterized by different combinations of NS with genital anomalies and pseudo-hermaphroditism [52, 53]. *WT1* is also known as the cause of sporadic and familial Wilms tumor [50], which is the most common solid tumor in childhood and represents a potential evolution of DDS. Genetic variations and clinical phenotypes allow an easy differentiation between DDS and FS since over 95% of DDS carry missense mutations in exons 8 and 9, while FS is more frequently associated with specific splice site mutations at IVS9, resulting in the presence or absence of a tri-peptide of lysine-threonine serine (KTS) in exon 9. In terms of renal involvement, DDS is predominantly characterized by diffuse mesangial sclerosis with early onset and rapid evolution to end stage renal failure, while FS usually presents slowly progressive FSGS, although rare variants with glomerular basement membrane involvement have been reported [54]. The real impact of *WT1* mutations in children with steroid resistant nephrotic syndrome (SRNS) has been recently evaluated by Ruf and colleagues [55] and in an extended cohort by Mucha and colleagues [56]. These studies showed an overall incidence of 7%, with a prevalence of FS over DDS phenotypes. It was striking that only one female out of eight with FS presented XY inversion and sexual anomalies that have long been considered a key sign of the syndrome. This finding strongly supports the need for the mutational analysis of *WT1* in children affected by NS with steroid resistance in order to avoid potentially harmful therapeutic interventions. An extension of screening studies to large cohorts of children and adults with SRNS is the natural consequence of the above findings. Aucella and colleagues [57] screened 115 patients with SRNS and 86 with steroid dependence (85 adults) for *WT1* mutations, with results that partially confirm the findings of Ruf and colleagues [55]. This study [57] also extends the basic histopathologic picture of FS to unusual renal findings, describing two cases with thinning of the basement membrane and with the presence of foam cells in the interstitium that appear to represent the prevalent early feature in FS patients.

### Pierson syndrome (OMIM#609049)

Pierson syndrome [58] is caused by mutations in the *LAMB2* gene that codes for laminin β2, a protein widely expressed in the glomerular basement membrane, retina, lens capsule and neuromuscular synapses [59]. The syndrome, characterized by microcoria and abnormal lens shape and cataract, is associated with NS that develops ‘in utero’ or within the first 3 months of life. The renal histopathologic lesion is DMS, and the outcome is usually a rapid progression to renal failure. In addition, patients surviving infancy develop blindness and severe neurological deficits [60]. The classical form is caused by truncating mutations of *LAMB2* [61]. Missense and non-truncating mutations of *LAMB2* are associated with a milder phenotype without microcoria or with minor structural eye anomalies, always with histological evidence of FSGS [62, 63]. Finally, *LAMB2* missense and truncating mutations have been reported in two cases presenting congenital NS without any ocular anomalies [64], suggesting that *LAMB2* may be implicated in inherited NS more frequently than expected.

### Schimke immuno-osseous dysplasia (OMIM#242900*)*

Schimke immuno-osseous dysplasia is an AR condition characterized by spondyloepiphyseal dysplasia with T-cell immunodeficiency and glomerulosclerosis [65]. Hypothyroidism and episodes of cerebral ischemia were found to be associated in a fraction of patients [66]. In 2002, Boerkoel and colleagues identified mutations of the *SMARCAL1* gene in 26 families with the disease, most of whom presented a severe phenotype [67]. *SMARCAL1* codes for a protein that participates in DNA remodeling after replication. Schimke immuno-osseous dysplasia is a clinically heterogeneous disease since severity ranges from a very early onset and death in the first years of life to milder forms that start in the first decade of life and survive beyond the second. In this disease, glomerulosclerosis evolves to end-stage renal failure requiring replacement treatment and renal transplantation.

### Action myoclonus–renal failure syndrome (OMIM#254900)

Action myoclonus–renal failure syndrome (AMRF) is an AR condition characterized by progressive myoclonus epilepsy associated with renal failure. Proteinuria is the hallmark and first symptom of the disease. Age at onset is between 15 and 20 years, and glomerulosclerosis with focal collapse is the usual histopathologic picture. Neurological symptoms, i.e. tremor, action myoclonus, seizures and ataxia, develop at a later stage and are produced by a characteristic deposition of lysosomal storage material in the brain. *SCARB2* is the gene whose mutations are responsible for the disease. It codes for a lysosomal integral membrane protein that has pleiotropic molecular functions. Action myoclonus–renal failure syndrome is now considered a storage disease due to alterations in lysosome functions and, like any other conditions involving lysosomes, it is mainly characterized by degenerative lesions of the brain [68].

### Nail–patella syndrome (OMIM#161200)

Nail–patella syndrome, also known as osteo-onychodysplasia, is an AD disorder classically producing dysplastic fingernails and hypoplastic or absent patella in association with renal defects that have been found in 40% of cases [69]. Glomerular changes involve both podocyte foot-process effacement [70] and thickening of the glomerular basement membrane as a result of the irregular deposition of fibrillar collagen type III [71]. The disease is caused by mutations of the *LMX1B* gene that codes for a transcription factor active at a very early stage of podocyte development [70]. The *Lmx1b* gene knockout mice present a retarded maturation of podocytes that do not develop foot-processes nor express major slit-diaphragm genes, such as podocin and nephrin. Alterations of the glomerular basement membrane and of the endothelial fenestration suggest a direct role of *LMX1B* at these sites [72].

## Galloway–Mowat syndrome (OMIM#251300)

The Galloway-Mowat syndrome is a rare condition originally described in 1968 in three sibs with the triad of congenital NS, microcephaly and hiatus hernia [73]. It is considered to be an AR disorder whose causative gene(s) are still unknown. Clinical features are generally represented by early onset NS with strict resistance to steroids and a poor prognosis [74]. Milder variants have also been reported, with the first renal symptom appearing after 10 years of age [75]. Renal pathology is also heterogeneous and varies from minimal changes to capillary proliferation, glomerulosclerosis and, in some cases, DMS [76].

## Mitochondrial cytopathies

### *tRNA*^*Leu(UUR)*^

Nephrotic syndrome may be part of a generalized defect induced by mutations in mitochondrial DNA (mtDNA). This condition preferentially affects the muscle and the nervous system but may also extend to the kidney. A typical example is the A3243G transition in the *tRNA*^*Leu(UUR)*^ gene that is classically associated with MELAS syndrome (mitochondrial myopathy, encephalopathy, lactic acidosis, stroke episodes and occasional tubular defects) [77]. This mutation has also been found to be associated with FSGS in patients without any other sign of MELAS [78]. The clinical phenotype in children with an isolated renal defect is characterized by variable proteinuria, from mild to severe, with resistance to steroids and slow progression to end stage renal failure.

### CoQ_10_ deficiency: *COQ2* and *PDSS2* (OMIM#607426)

Congenital deficiency of coenzyme Q_10_ (CoQ_10_) includes a group of AR anomalies that are primarily characterized by neurological and muscular symptoms [79, 80] but may also present with isolated NS [81]. They are produced by mutations of genes coding for enzymes of the CoQ_10_ pathway, only a part of which have been identified. *COQ2* is one of these genes. It codes for para-hydroxybenzoate-polyprenyl-transferase (EC 2.5.1.39), which catalyzes prenylation of para-hydroxybenzoate [82]. Mutations of *COQ2* have been reported in association with isolated NS in two children and/or as part of a multi-organ defect producing progressive encephalopathy with muscular hypotonia and optic nerve atrophy in two other children. Severe NS with rapid progression to terminal renal failure is a predominant clinical feature. Renal histopathology was characterized by collapsing glomerulopathy and severe extracapillary proliferation in two cases, while FSGS was found in two siblings. Ultrastructural findings showed dysmorphic mitochondria with electron-lucent bodies in all cases and oncocyte-like aspects in a few. Biochemical analysis showed a decreased complex [II + III] activity in the renal cortex and skeletal muscle [81].

*PDSS2* is the second gene implicated in CoQ_10_ deficiency syndrome. It codes for decaprenyl diphosphate synthase, the first enzyme of the CoQ_10_ biosynthetic pathway [83]. The first and unique case with compound heterozygous mutations of *PDSS2* described to date presented [35] with severe Leigh syndrome characterized by seizures, hypotonia, cortical blindness and NS [83]. Biochemical analysis performed in muscles and fibroblasts demonstrated decreased complex [II + III] activity and a severe defect in decaprenyl diphosphate synthase.

Literature data indicate that children with congenital NS and encephalopathy should be screened for primary defect in CoQ_10_ biosynthesis. Cases with isolated renal defect, i.e. collapsing glomerulopathy or crescentic glomerulosclerosis, should also be screened. Molecular analysis of both *COQ2* and *PDSS2* is now available in several laboratories and should be performed in those patients with the above phenotypes. Therapy with coenzyme Q_10_ should be started very early since it may completely resolve NS [84] and actually represents the unique example of successful therapy for mitochondrial defects.

## Drug-responsive variants of NS

Steroid-sensitive NS (SSNS) may occasionally occur in association with mutations of some of the genes mentioned above, but a good response to therapy is, in general, considered to be a rare event [42, 85, 86]. Two examples of inherited conditions responsive to steroids are the few cases with mild mutations of nephrin recently reported by Philippe and colleagues [42] and the unique girl carrying homozygous truncating *PLCE1* mutations [35].

Familial aggregates and kindred with SSNS in geographical isolates have been described, suggesting a potential genetic origin. Very little data on molecular genetics of SSNS are available in the literature. A genome-wide scan for linkage has been recently conducted in 11 families with an AR trait, one of which was a consanguineous kindred from Germany. The first locus for SSNS was identified on chromosome 2p12–13.2 in an interval of 3.6 Mb that contains approximately 60 known genes and 25 predicted genes [87]. The characterization of the first gene involved in NS responsive to steroids would represent a critical step in the definition of the underlying mechanism of non-inherited forms of the disease.

## Recurrence of disease after renal transplantation

The underlying factors for post-transplant recurrence of proteinuria in FSGS remains an enigma. Patients with the sporadic variety of FSGS who have known homozygous or complex heterozygous traits present a low recurrence rate [88], and this appears to be a logical consequence of the fact that proteinuria is originally due to the inherited defect in the native organ while the grafted kidney is normal. This aspect has mostly been considered in patients with *NPHS2* mutations. An original report by Bertelli and colleagues [89] described two patients with homozygous or compound heterozygous mutations of *NPHS2* (22% of those who received a renal graft), and in both cases the episode had a mild clinical impact with favorable outcome. Another two cases of recurrent FSGS have been recently described: one had the *NPHS2* mutation [90] and the other had the *WT1* mutation (IVS9 + 4C>T) and Frasier syndrome [91]. More recently, Sanna-Cherchi and colleagues [92] described a pedigree with AD FSGS of unknown origin in which the index case presented post-transplant recurrence of severe proteinuria and MCD. The identification of families with AD transmission and recurrence of disease has provided the chance to identify extra-renal factors involved in the pathogenesis of sporadic forms of FSGS. We now have the opportunity to consider post-transplant recurrence in a larger population and to compare the original data by Bertelli and colleagues [89] with other study cohorts [18, 19]. Collectively (including the study by Bertelli), data are available for 65 carriers of homozygous or compound heterozygous *NPHS2* mutations, and the post-transplant recurrence of proteinuria was documented in five of these (7.7%). It is worth reporting that one child with the homozygous p.L347X mutation described by Ruf and colleagues [18, 93] and another one with homozygous p.R138X described by Weber and colleagues [19] received the kidney from their mother, who was an obligatory healthy heterozygous carrier. The first child had recurrence of proteinuria on day 7 from the graft and responded within 1 week to plasmapheresis. The second child presented nephrotic range proteinuria 2 years after the allograft. While the low number of patients with a single *NPHS2* mutation does not allow any definite conclusion to be drawn, these results strongly suggest that the rate of recurrence in patients with *NPHS2* homozygous or compound heterozygous mutations is low. Grafts from obligatory carriers of *NPHS2* mutations, such as parents of affected individuals, should be avoided because of the higher risk of recurrence of FSGS.

**Questions**:

(Answers appear following the reference list)

1. Which is the most frequent mode of inheritance of familial NS?
  - AD
  - AR
  - X-linked dominant
  - All
2. Genes involved in NS code most frequently for proteins of
  - glomerular basement membrane
  - podocytes
  - tubular epithelia
  - mesangial cells
3. *TRCP6* mutations are associated with congenital NS
  - Yes, always
  - Often
  - Not yet
  - With Finnish nephropathy
4. *PLCE1* usually causes DMS
  - In most cases
  - The association has never been reported
  - Is more frequently associated with FSGS
  - Is usually associated with hermaphroditism
5. *NPHS1* truncating mutation (Fin_major_) is prevalent in
  - Southern Europe
  - France
  - Finland
  - Only in Asia
6. Denys-Drash Syndrome is allelic with
  - Frasier syndrome
  - Leigh syndrome
  - *NPHS1*
  - *NPHS2*
7. Which is the gene for Familial Steroid Sensitive NS?
  - *LAMB2*
  - *ACTN4*
  - *WT1*
  - Never reported

**Answers**

b) AR
b) Podocytes
c) Not yet
a) In most cases
c) Finland
a) Frasier syndrome
d) Never reported
